# Nanostructures from Synthetic Genetic Polymers

**DOI:** 10.1002/cbic.201600136

**Published:** 2016-04-20

**Authors:** Alexander I. Taylor, Fabienne Beuron, Sew‐Yeu Peak‐Chew, Edward P. Morris, Piet Herdewijn, Philipp Holliger

**Affiliations:** ^1^ Medical Research Council Laboratory of Molecular BiologyFrancis Crick AvenueCambridgeCB2 0QHUK; ^2^ Department of Biology/Centre for Applied Synthetic BiologyConcordia University7141 Rue SherbrookeMontrealH4B 1R6Canada; ^3^ Division of Structural BiologyThe Institute of Cancer ResearchChester Beatty Laboratories)237 Fulham RoadLondonSW3 6JBUK; ^4^ Rega InstituteKU LeuvenMinderbroedersstraat 103000LeuvenBelgium; ^5^ Institute of Systems and Synthetic BiologyUniversité Evry5 rue Henri Desbrueres91030Evry CedexFrance

**Keywords:** chemical biology, DNA nanotechnology, electron microscopy, self-assembly, xeno nucleic acids (XNAs)

## Abstract

Nanoscale objects of increasing complexity can be constructed from DNA or RNA. However, the scope of potential applications could be enhanced by expanding beyond the moderate chemical diversity of natural nucleic acids. Here, we explore the construction of nano‐objects made entirely from alternative building blocks: synthetic genetic polymers not found in nature, also called xeno nucleic acids (XNAs). Specifically, we describe assembly of 70 kDa tetrahedra elaborated in four different XNA chemistries (2′‐fluro‐2′‐deoxy‐ribofuranose nucleic acid (2′F‐RNA), 2′‐fluoroarabino nucleic acids (FANA), hexitol nucleic acids (HNA), and cyclohexene nucleic acids (CeNA)), as well as mixed designs, and a ∼600 kDa all‐FANA octahedron, visualised by electron microscopy. Our results extend the chemical scope for programmable nanostructure assembly, with implications for the design of nano‐objects and materials with an expanded range of structural and physicochemical properties, including enhanced biostability.

Nucleic acids are molecules of astonishing versatility. In addition to their well‐known roles in genetic information storage and propagation, they can act as sensors,^1^ catalysts,^2^ and regulators of gene expression.^3^ Furthermore, longer DNA and RNA polymers can fold into highly complex three‐dimensional (3 D) structures.^4^, ^5^ Together with the well‐understood Watson–Crick self‐association rules, this has enabled the use of nucleic acids (initially DNA, but increasingly RNA) as a scaffold for construction of nanoscale objects and devices,^6^, ^7^, ^8^ including polyhedra and lattices,^9^, ^10^ 2 D and 3 D origami objects,^11^ and DNA brick structures.^12^ Such programmable, self‐assembling DNA and RNA nanostructures have shown potential for a wide variety of applications,^13^ including sensing,^14^ in vivo computation,^15^ siRNA delivery,^16^, ^17^ encapsulation and release of therapeutic cargo,^18^, ^19^, ^20^ organisation of biosynthetic enzymes on supramolecular assemblies,^21^, ^22^ or even formation of membrane‐spanning pores.^23^ However, the comparatively low biostability^24^ and immunogenicity^25^ of natural nucleic acids, together with limited chemical diversity and constraints on architecture and self‐assembly dynamics,^26^ restrict the scope of potential applications of DNA and RNA nanotechnology. Although some improvements might be gained though novel design strategies^29^ or sporadic incorporation of DNA modifications,^30^, ^31^, ^32^ we reasoned that a broad expansion of the range of nucleic acid chemistries available for nanotechnology could allow designs to exploit physicochemical properties beyond those of natural polymers.

Here, we report the construction of nanotechnology objects with wholesale replacement of natural nucleic acid strands with unnatural analogues, specifically synthetic genetic polymers, also known as xeno nucleic acids (XNAs). XNAs have previously been shown to be capable of XNA–XNA duplex formation^33^, ^34^ and can fold into 3 D structures, forming ligands (aptamers)^28^, ^35^, ^36^ and catalysts (XNAzymes).^37^ This offers a range of divergent structures and properties^38^ of potential benefit to biotechnology and medicine.^39^ However, de novo design in the absence of detailed knowledge on XNA structural and conformational parameters is challenging. Hybrid nanostructures based on DNA designs have previously been demonstrated to retain overall architecture, despite invasion by strands composed of, inter alia, peptide nucleic acids (PNA)^41^, ^42^, ^43^ or phosphorothioate DNA (PS‐DNA).^32^ Furthermore, a functional Phi29 DNA‐packing motor can be assembled with partial substitution of RNA components with 2′‐fluro‐2′‐deoxy‐ribofuranose nucleic acid (2′F‐RNA).^44^ These results indicate that, at least in some cases, structures and folding topologies can be maintained when using artificial polymers. We therefore sought to explore the potential for well‐established DNA nanotechnology designs to form self‐assembling nanostructures entirely composed of XNA strands. Using a series of engineered polymerases,^28^, ^37^, ^45^ we first synthesised fully XNA‐substituted analogues of the four 55‐mer strand components of the classic Turberfield DNA tetrahedron,^27^ elaborated in four different XNA chemistries: 2′F‐RNA, 2′‐fluoroarabino nucleic acids (FANA),^46^ hexitol nucleic acids (HNA), and cyclohexene nucleic acids (CeNA),^33^ verified by mass spectrometry (Figure S1 in the Supporting Information). Despite their known structural and conformational differences,^38^ all four XNA chemistries formed tetrahedra under physiological conditions in a single‐step reaction, as determined by a non‐denaturing gel electrophoresis mobility shift assay (EMSA; Figure 1). Indeed, strands composed of 2′F‐RNA and FANA (which preferentially adopt A‐form and B‐form duplexes, respectively^34^, ^47^) were even able to substitute for DNA strands in mixed‐chemistry structures (Figure S2), suggesting an ability of robust designs to overcome conformational preferences. To further verify the correct assembly and global structures of the assembled XNA tetrahedra, we coupled gold nanoparticles (AuNPs) to each vertex and imaged the resulting nano‐objects by transmission electron microscopy (TEM, Figure 2) according to a simple quasi‐3 D imaging method.^40^ Intact tetrahedra could be distinguished as 3 D structures from 2 D partially assembled versions in non‐annealed samples (Figure S3) by examining the relative parallax motion of AuNPs as sample grids were tilted.

**Figure 1:**
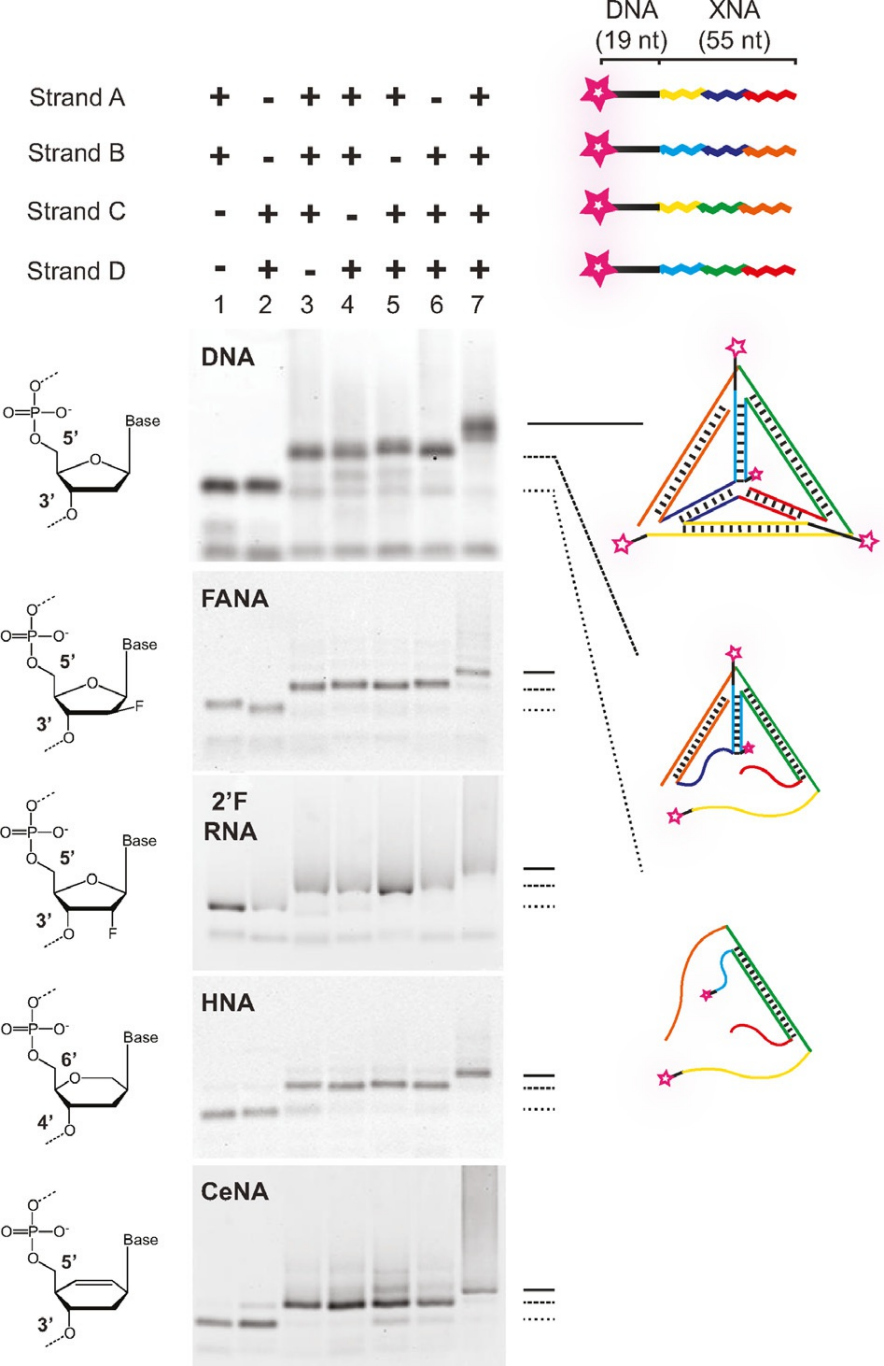
Single‐step self‐assembly of XNA tetrahedra. A tetrahedron designed to form from four single‐stranded 55‐mer DNA oligonucleotides^27^ (strands A–D) containing regions of complementarity (corresponding colours) can be assembled from analogous strands composed of a variety of XNAs^28^ Tris**⋅**HCl buffer (10 mm, pH 8.0), containing NaCl (125 mm) and EDTA (1 mm). DNA was folded in Tris**⋅**HCl buffer (10 mm, pH 8.0), containing EDTA (1 mm) and MgCl_2_ (10 mm). For all chemistries shown, absence of any one or more strands (lanes 1–6) caused a shift in mobility during agarose gel electrophoresis (2 %, 0.5× TBE) compared with all four components (lane 7).

**Figure 2:**
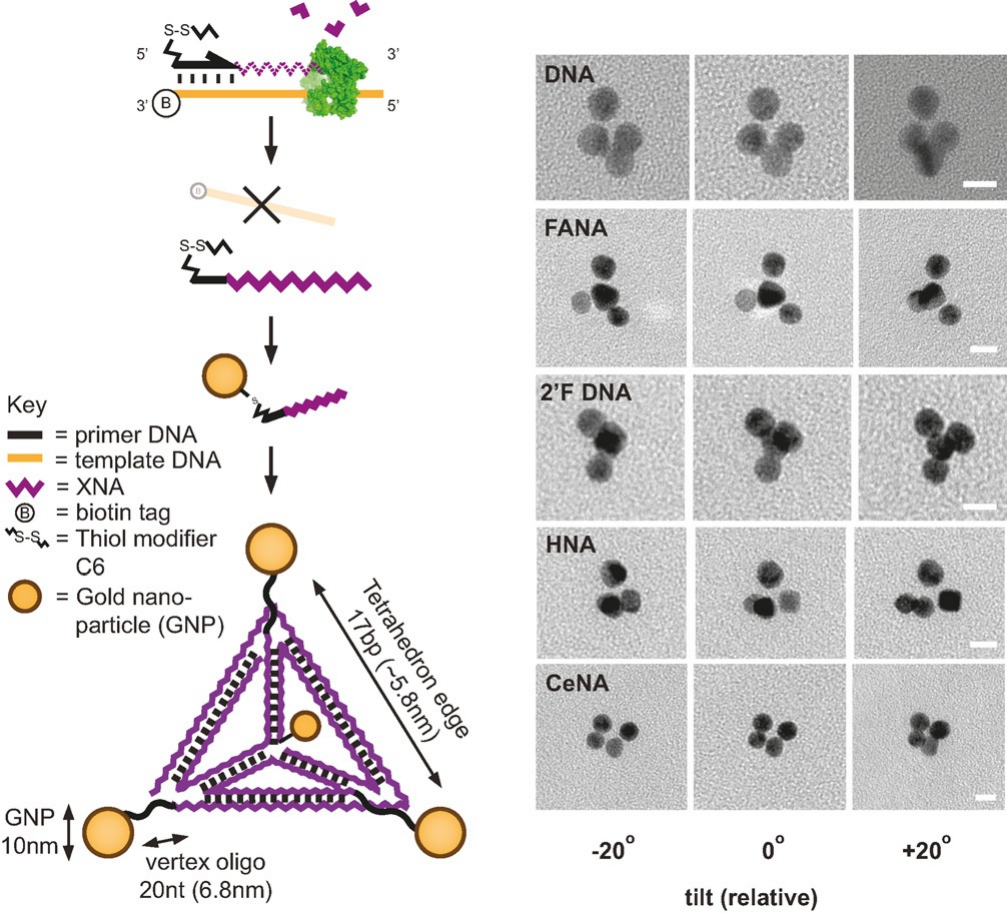
Verification of XNA tetrahedra structure by TEM. XNA strands were prepared by using 5′‐thiol‐modified DNA primers to allow conjugation to AuNPs. The 3 D structure of tetrahedra assembled from AuNP‐labelled strands was confirmed by TEM at different tilting angles.^40^

To demonstrate advantageous XNA‐specific properties, we incubated tetrahedra composed of DNA or HNA in serum‐containing cell culture media at 37 °C and examined degradation by agarose gel electrophoresis (Figure S4). Although assembly into tetrahedra^48^ or more complex designs^29^ has been observed to offer some degree of protection by itself, DNA tetrahedra were fully degraded after 1–2 days, whereas HNA tetrahedra remained intact even after 8 days.

Many DNA nanostructures employ an origami‐like strategy in which a long polymer is folded into a 3 D shape through intramolecular interactions, defined by short DNA staple strands.^11^ In order to examine whether XNAs would be capable of origami folding, we synthesised the 1.7 kb main chain and the five 40‐mer staple strands that comprise a designed DNA octahedron^49^ by using exclusively FANA chemistry (Figure S5). The DNA octahedron has a branched‐tree design held together by paranemic and double‐strand crossover junctions (see ref. 49 for full details) that can be induced to fold into the octahedron upon addition of magnesium counterions (Mg^2+^). The FANA octahedron displayed essentially identical Mg^2+^‐dependent folding behaviour compared to the DNA version, as judged by EMSA (Figure 3).

**Figure 3:**
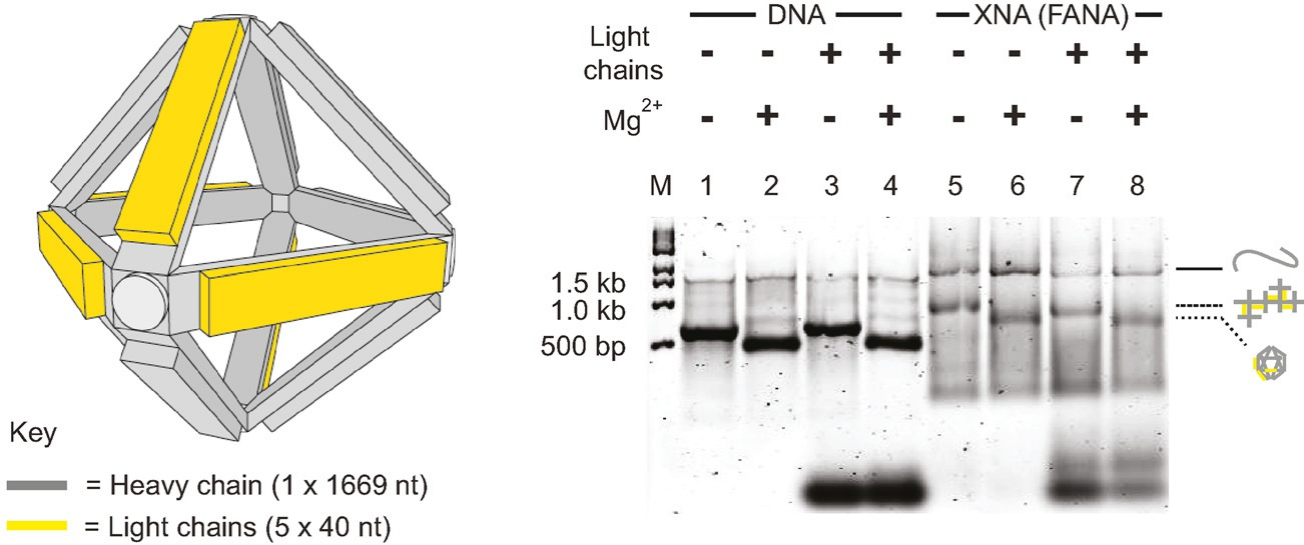
Assembly of an XNA octahedron. The components of the Shi et al. DNA octahedron^49^ (grey and yellow) were synthesised by using FANA nucleotides. Gel‐shift patterns of DNA (lanes 1–4) and FANA (lanes 5–8) versions were compared after folding in the presence or absence of Mg^2+^ (even or odd numbered lanes, respectively) and DNA (lanes 3 and 4) or FANA (lanes 7 and 8) light chains on 2 % agarose gels run at 100 V and 4 °C, in Tris buffer (45 mm, pH 8.0) with boric acid (45 mm) and with or without MgCl_2_ (2 mm) and gel star stain. FANA octahedron assembly is evident by an increase in mobility comparable to that of the DNA version.

In order to verify assembly and examine the effect of FANA chemistry on octahedron topology and structure, we visualised all‐FANA octahedra by using negative‐stain TEM (Figure 4). We readily identified structures resembling TEM images of DNA octahedra^49^ and were able to generate a 3 D model by single‐particle reconstruction at ∼30 Å resolution. This revealed a 180 Å cage‐like structure consistent with the overall design, albeit with potential alternative conformations (Figure S6) and deviating from a regular octahedron by curvature of the twelve struts comprising the octahedron edges. This might be due to the structural differences between FANA and DNA, such as the increased rigidity and non‐canonical O4′‐endo (east) conformation of the fluorinated arabinose sugar and enhanced inter‐residual interactions,^34^ whose effect on the architecture of crossover junctions has yet to be studied.

**Figure 4:**
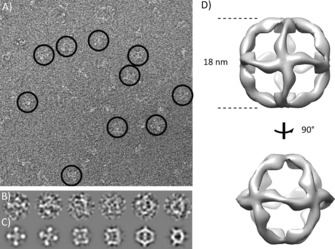
Imaging of XNA octahedra by TEM. A) Wide‐field view of negatively stained XNA (FANA) octahedra. Typical particles adopting different orientations on the carbon support film are circled. B) Selection of single particles and C) corresponding reprojections of the 3 D map obtained from single‐particle reconstruction in RELION (Supporting Information). D) Two surface views of the 3 D model.

In summary, we describe the first elaboration of nucleic acid nanostructures using entirely synthetic XNA building blocks. Our work shows that, unlike DNA and RNA ligands and catalysts obtained by in vitro evolution, at least some DNA designs can be converted into broadly equivalent XNA nanostructures. It is too early to predict if this will be a general finding or be restricted to exceptionally flexible and robust designs. Indeed, even within the designs explored herein, differences between the structures of DNA and FANA octahedra were evident. In the case of the tetrahedron, we observed that designs with two unpaired nucleotide vertex hinges^27^ folded with much higher yields than those comprising single residue hinges^50^ (data not shown), presumably because this more constrained design was less able to accommodate the divergent structural preferences of these XNAs. As with RNA,^51^ a fuller realisation of the potential of novel construction materials for nanotechnology will require a more detailed investigation of the chemistry‐specific structural and conformational parameters, for which current knowledge is sparse. The XNA nanostructures described herein present clear opportunities to derive such parameters in the future, for example, through higher resolution electron microscopy structures.

The wider introduction of XNA chemistries into the design and assembly of nanotechnology objects thus promises not only an expansion of chemical diversity beyond DNA and RNA but of structural and physicochemical parameters relevant to a variety of applications, from medicine to materials science.

## Supporting information

As a service to our authors and readers, this journal provides supporting information supplied by the authors. Such materials are peer reviewed and may be re‐organized for online delivery, but are not copy‐edited or typeset. Technical support issues arising from supporting information (other than missing files) should be addressed to the authors.

Supplementary

## References

[1] A. Serganov , E. Nudler , Cell 2013, 152, 17–24. 23332744 10.1016/j.cell.2012.12.024 PMC4215550

[2] R. R. Breaker , G. F. Joyce , Chem. Biol. 2014, 21, 1059–1065. 25237854 10.1016/j.chembiol.2014.07.008 PMC4171699

[3] T. R. Cech , J. A. Steitz , Cell 2014, 157, 77–94. 24679528 10.1016/j.cell.2014.03.008

[4] P. Nissen , J. Hansen , N. Ban , P. B. Moore , T. A. Steitz , Science 2000, 289, 920–930. 10937990 10.1126/science.289.5481.920

[5] S. M. Fica , M. A. Mefford , J. A. Piccirilli , J. P. Staley , Nat. Struct. Mol. Biol. 2014, 21, 464–471. 24747940 10.1038/nsmb.2815 PMC4257784

[6] F. Zhang , J. Nangreave , Y. Liu , H. Yan , J. Am. Chem. Soc. 2014, 136, 11198–11211. 25029570 10.1021/ja505101a PMC4140475

[7] W. W. Grabow , L. Jaeger , Acc. Chem. Res. 2014, 47, 1871–1880. 24856178 10.1021/ar500076k

[8] M. R. Jones , N. C. Seeman , C. A. Mirkin , Science 2015, 347, 1260901. 25700524 10.1126/science.1260901

[9] N. C. Seeman , J. Theor. Biol. 1982, 99, 237–247. 6188926 10.1016/0022-5193(82)90002-9

[10] A. Chworos , I. Severcan , A. Y. Koyfman , P. Weinkam , E. Oroudjev , H. G. Hansma , L. Jaeger , Science 2004, 306, 2068–2072. 15604402 10.1126/science.1104686

[11] P. W. K. Rothemund , Nature 2006, 440, 297–302. 16541064 10.1038/nature04586

[12] Y. Ke , L. L. Ong , W. M. Shih , P. Yin , Science 2012, 338, 1177–1183. 23197527 10.1126/science.1227268 PMC3843647

[13] P. K. Lo , K. L. Metera , H. F. Sleiman , Curr. Opin. Chem. Biol. 2010, 14, 597–607. 20869905 10.1016/j.cbpa.2010.08.002

[14] H. Pei , X. Zuo , D. Pan , J. Shi , Q. Huang , C. Fan , NPG Asia Mater. 2013, 5, e51.

[15] Y. Amir , E. Ben-Ishay , D. Levner , S. Ittah , A. Abu-Horowitz , I. Bachelet , Nat. Nanotechnol. 2014, 9, 353–357. 24705510 10.1038/nnano.2014.58 PMC4012984

[16] A. S. Walsh , H. Yin , C. M. Erben , M. J. A. Wood , A. J. Turberfield , ACS Nano 2011, 5, 5427–5432. 21696187 10.1021/nn2005574

[17] K. A. Afonin , M. Viard , A. Y. Koyfman , A. N. Martins , W. K. Kasprzak , M. Panigaj , R. Desai , A. Santhanam , W. W. Grabow , L. Jaeger , E. Heldman , J. Reiser , W. Chiu , E. O. Freed , B. A. Shapiro , Nano Lett. 2014, 14, 5662–5671. 25267559 10.1021/nl502385k PMC4189619

[18] C. M. Erben , R. P. Goodman , A. J. Turberfield , Angew. Chem. Int. Ed. 2006, 45, 7414–7417; 10.1002/anie.200603392 17086586

[19] S. M. Douglas , I. Bachelet , G. M. Church , Science 2012, 335, 831–834. 22344439 10.1126/science.1214081

[20] Y.-X. Zhao , A. Shaw , X. Zeng , E. Benson , A. M. Nyström , B. Högberg , ACS Nano 2012, 6, 8684–8691. 22950811 10.1021/nn3022662

[21] J. Fu , Y. R. Yang , A. Johnson Buck , M. Liu , Y. Liu , N. G. Walter , N. W. Woodbury , H. Yan , Nat. Nanotechnol. 2014, 9, 531–536. 24859813 10.1038/nnano.2014.100

[22] G. Sachdeva , A. Garg , D. Godding , J. C. Way , P. A. Silver , Nucleic Acids Res. 2014, 42, 9493–9503. 25034694 10.1093/nar/gku617 PMC4132732

[23] M. Langecker , V. Arnaut , T. G. Martin , J. List , S. Renner , M. Mayer , H. Dietz , F. C. Simmel , Science 2012, 338, 932–936. 23161995 10.1126/science.1225624 PMC3716461

[24] J. Hahn , S. F. J. Wickham , W. M. Shih , S. D. Perrault , ACS Nano 2014, 8, 8765–8775. 25136758 10.1021/nn503513p PMC4174095

[25] S. Surana , A. R. Shenoy , Y. Krishnan , Nat. Nanotechnol. 2015, 10, 741–747. 26329110 10.1038/nnano.2015.180 PMC4862568

[26] A. V. Pinheiro , D. Han , W. M. Shih , H. Yan , Nat. Nanotechnol. 2011, 6, 763–772. 22056726 10.1038/nnano.2011.187 PMC3334823

[27] R. P. Goodman , R. M. Berry , A. J. Turberfield , Chem. Commun. 2004, 1372–1373. 10.1039/b402293a 15179470

[28] V. B. Pinheiro , A. I. Taylor , C. Cozens , M. Abramov , M. Renders , S. Zhang , J. C. Chaput , J. Wengel , S. Y. Peak-Chew , S. H. McLaughlin , P. Herdewijn , P. Holliger , Science 2012, 336, 341–344. 22517858 10.1126/science.1217622 PMC3362463

[29] V. Cassinelli , B. Oberleitner , J. Sobotta , P. Nickels , G. Grossi , S. Kempter , T. Frischmuth , T. Liedl , A. Manetto , Angew. Chem. Int. Ed. 2015, 54, 7795–7798; 10.1002/anie.201500561 25980669

[30] J. W. Conway , C. K. McLaughlin , K. J. Castor , H. Sleiman , Chem. Commun. 2013, 49, 1172–1174. 10.1039/c2cc37556g 23287884

[31] J. P. Peters , S. P. Yelgaonkar , S. G. Srivatsan , Y. Tor , L. J. Maher , Nucleic Acids Res. 2013, 41, 10593–10604. 24013560 10.1093/nar/gkt808 PMC3905893

[32] J. R. Burns , E. Stulz , S. Howorka , Nano Lett. 2013, 13, 2351–2356. 23611515 10.1021/nl304147f

[33] P. Herdewijn , Chem. Biodiversity 2010, 7, 1–59. 10.1002/cbdv.200900185 20087996

[34] N. Martin-Pintado , M. Yahyaee-Anzahaee , R. Campos-Olivas , A. M. Noronha , C. J. Wilds , M. J. Damha , C. Gonzalez , Nucleic Acids Res. 2012, 40, 9329–9339. 22798499 10.1093/nar/gks672 PMC3467067

[35] I. Alves Ferreira-Bravo , C. Cozens , P. Holliger , J. J. DeStefano , Nucleic Acids Res. 2015, 43, 9587–9599. 26476448 10.1093/nar/gkv1057 PMC4751925

[36] H. Yu , S. Zhang , J. C. Chaput , Nat. Chem. 2012, 4, 183–187. 22354431 10.1038/nchem.1241

[37] A. I. Taylor , V. B. Pinheiro , M. J. Smola , A. S. Morgunov , S. Peak-Chew , C. Cozens , K. M. Weeks , P. Herdewijn , P. Holliger , Nature 2015, 518, 427–430. 25470036 10.1038/nature13982 PMC4336857

[38] I. Anosova , E. A. Kowal , M. R. Dunn , J. C. Chaput , W. D. Van Horn , M. Egli , Nucleic Acids Res. 2016, 44, 1007–1021. 26673703 10.1093/nar/gkv1472 PMC4756832

[39] A. I. Taylor , S. Arangundy-Franklin , P. Holliger , Curr. Opin. Chem. Biol. 2014, 22, 79–84. 25285754 10.1016/j.cbpa.2014.09.022

[40] A. Stern , D. Rotem , I. Popov , D. Porath , J. Phys. Condens. Matter 2012, 24, 164203. 22465965 10.1088/0953-8984/24/16/164203

[41] T. Yamazaki , Y. Aiba , K. Yasuda , Y. Sakai , Y. Yamanaka , A. Kuzuya , Y. Ohya , M. Komiyama , Chem. Commun. 2012, 48, 11361–11363. 10.1039/c2cc36358e 23073563

[42] J. D. Flory , T. Johnson , C. R. Simmons , S. Lin , G. Ghirlanda , P. Fromme , Artif. DNA PNA XNA 2014, 5(3), 1–8. 10.4161/1949095X.2014.992181 PMC5329897 25760314

[43] R. O. Pedersen , J. Kong , C. Achim , T. H. LaBean , Molecules 2015, 20, 17645–17658. 26404232 10.3390/molecules200917645 PMC6331967

[44] J. Liu , S. Guo , M. Cinier , L. S. Shlyakhtenko , Y. Shu , C. Chen , G. Shen , P. Guo , ACS Nano 2011, 5, 237–246. 21155596 10.1021/nn1024658 PMC3026857

[45] C. Cozens , V. B. Pinheiro , A. Vaisman , R. Woodgate , P. Holliger , Proc. Natl. Acad. Sci. USA 2012, 109, 8067–8072. 22566643 10.1073/pnas.1120964109 PMC3361454

[46] C. J. Wilds , M. J. Damha , Nucleic Acids Res. 2000, 28, 3265–3625. 10.1093/nar/28.18.3625 PMC110742 10982885

[47] P. S. Pallan , E. M. Greene , P. A. Jicman , R. K. Pandey , M. Manoharan , E. Rozners , M. Egli , Nucleic Acids Res. 2011, 39, 3482–3495. 21183463 10.1093/nar/gkq1270 PMC3082899

[48] J.-W. Keum , H. Bermudez , Chem. Commun. 2009, 0, 7036–7038. 10.1039/b917661f 19904386

[49] W. M. Shih , J. D. Quispe , G. F. Joyce , Nature 2004, 427, 618–621. 14961116 10.1038/nature02307

[50] T. Kato , R. P. Goodman , C. M. Erben , A. J. Turberfield , K. Namba , Nano Lett. 2009, 9, 2747–2750. 19492821 10.1021/nl901265n

[51] H. Zuo , S. Wu , M. Li , Y. Li , W. Jiang , C. Mao , Angew. Chem. Int. Ed. 2015, 54, 15118–15121; 10.1002/anie.201507375 26457993

