# Supplementary material for: Nanostructures from Synthetic Genetic Polymers
Source: Chembiochem. 2016 Apr 20;17(12):1107–10. doi: 10.1002/cbic.201600136 (PMC4973672; doi:10.1002/cbic.201600136)
Supplement: Supplementary file 1 — Supplementary [file CBIC-17-1107-s001.pdf]

CHEMBIOCHEM

Supporting Information

Nanostructures from Synthetic Genetic Polymers

Alexander I. Taylor,*[a, b] Fabienne Beuron,[c] Sew-Yeu Peak-Chew,[a] Edward P. Morris,[c] Piet Herdewijn,[d, e] and Philipp Holliger*[a]

cbic_201600136_sm_miscellaneous_information.pdf

Table of Contents

Supplementary Data

Figure S1: Mass spectrometry of XNA oligonucleotides 1

Figure S2: Mixed-chemistry nanostructures 2

Figure S3: Quasi-3D TEM imaging of incompletely formed XNA tetrahedra 3

Figure S4: Stability of an XNA tetrahedron in serum-containing cell culture medium 4

Figure S5: Synthesis of DNA and XNA versions of the 1.7kb octahedron heavy chain 5

Figure S6: Alternative XNA (FANA) octahedron models 6

Table S1. Oligonucleotide sequences (5' -> 3') used for nanostructure synthesis 7

Experimental Section 9

Supplemental References 12

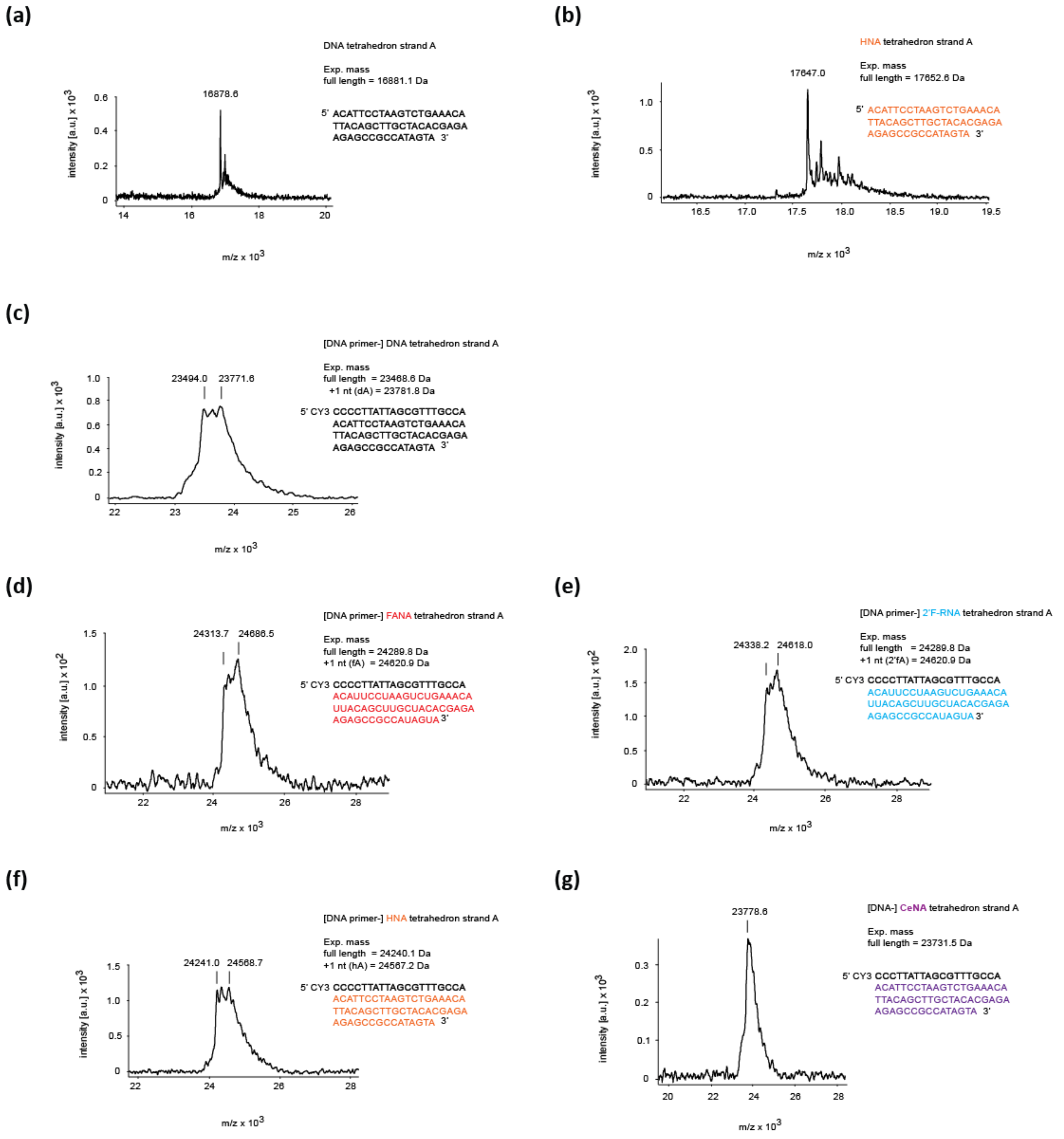

Figure S1: Mass spectrometry of XNA oligonucleotides.

MALDI-ToF mass spectra of tetrahedron component strands. **a**) DNA and **b**) HNA strands used to construct tetrahedra lacking vertex oligos (see Fig S4). Oligos used to construct DNA-tagged tetrahedra, composed of **c**) DNA, **d**) FANA, **e**) 2'F-RNA, **f**) HNA and **g**) CeNA. Mass spectra of DNA and all four XNA chemistries are consistent with expected full length products, or full length with single additions through extendase activity.

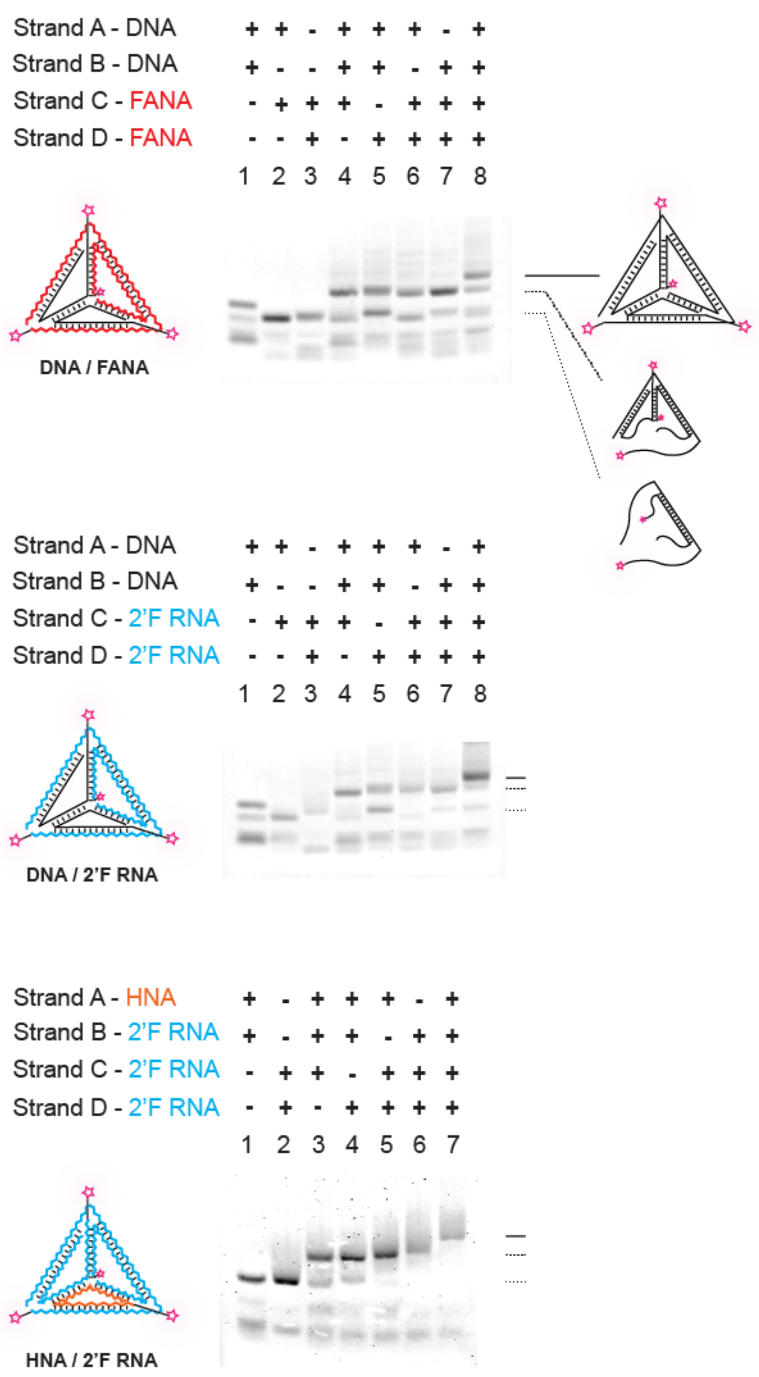

Figure S2: Mixed-chemistry nanostructures.

Component strands of DNA tetrahedra[1] can be substituted with equivalents composed of FANA or 2'F-RNA, yielding composite nanostructures, verified by non-denaturing agarose gel electrophoresis (3%, 0.5X TBE). HNA (and CeNA) versions of any of the four strands could not be combined with DNA; however, composite tetrahedra could be formed with 2'F-RNA and an HNA version of strand A.

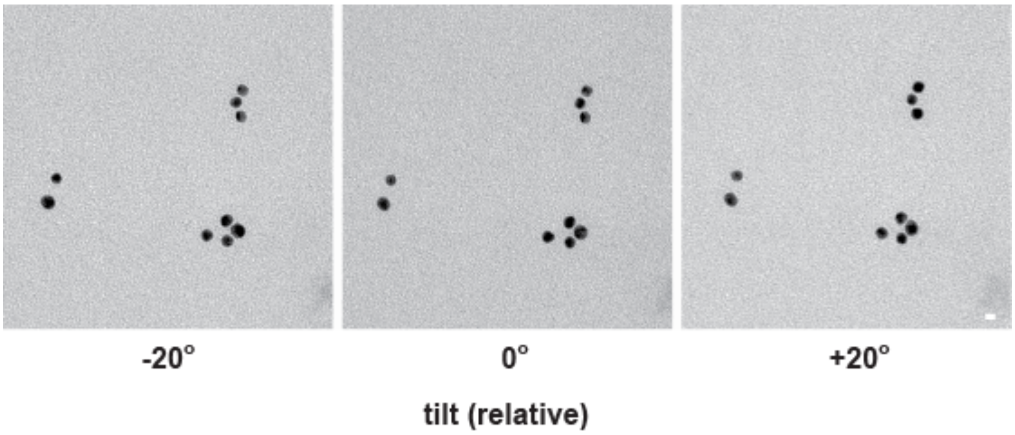

Figure S3: Quasi-3D TEM imaging of incompletely formed XNA tetrahedra.

An example of an unfolded four-strand structure (bottom right in each panel) or structures composed of only two (left in each panel) or three (top right in each panel) of the component strands, imaged by TEM in a sample of non-annealed, unpurified GNP-labeled HNA tetrahedra. In contrast with the 3D structures shown in Fig. 2, titling these objects shows little or no parallax motion, revealing that they are 2D objects in the same plane[2].

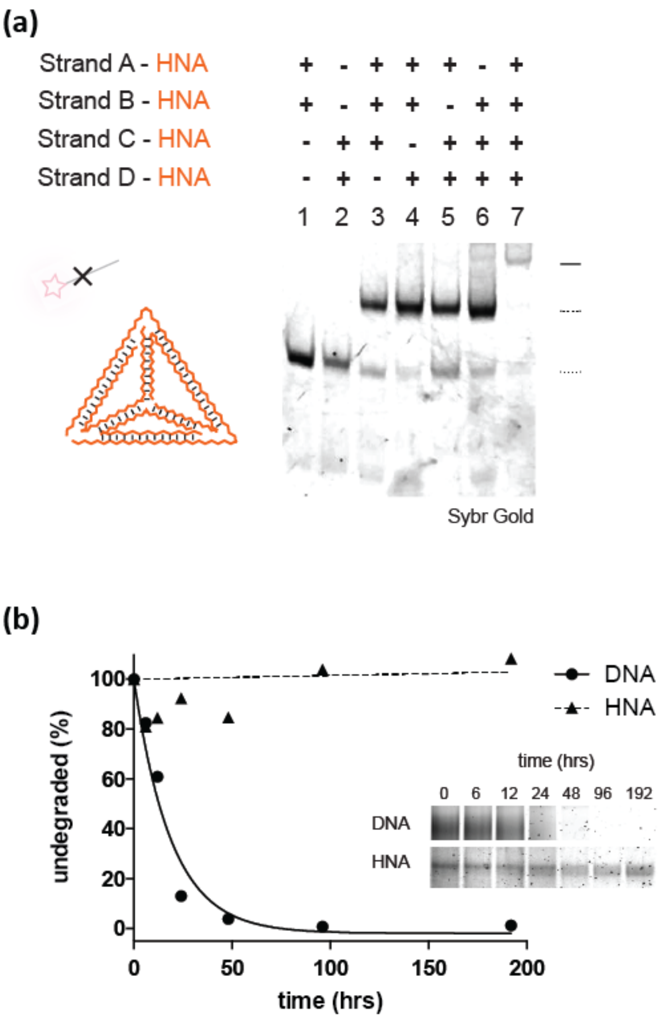

Figure S4: Stability of an XNA tetrahedron in serum-containing cell culture medium.

a) HNA strands (primed with RNA oligonucleotides, which were subsequently removed by alkaline hydrolysis) are used to assemble tetrahedra composed entirely of HNA, as verified by non-denaturing PAGE.
b) Degradation (%), as judged by densitometry of samples electrophoresed on non-denaturing agarose gel (3%, 0.5X TBE, stained with GelStar)(inset), is plotted vs. time for all-DNA tetrahedra (black circles) and all-HNA tetrahedra (black triangles). HNA tetrahedra, unlike DNA equivalents, are not degraded but remain stable during prolonged incubation at 37 ^{o}C in Hank's Balanced Salt Solution (HBSS) supplemented with 10 % FCS.

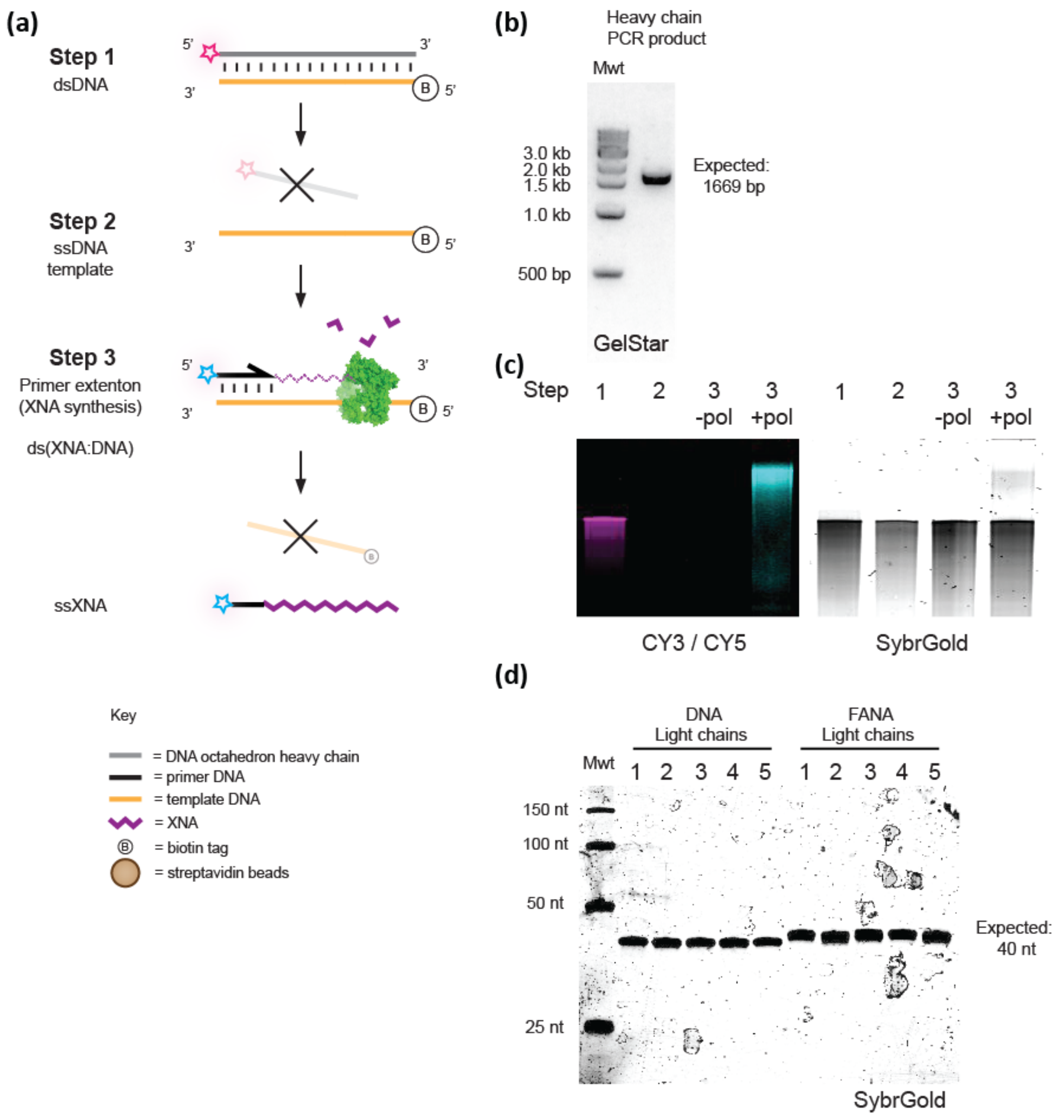

Figure S5: Synthesis of DNA and XNA versions of the 1.7kb octahedron heavy chain.

a) In step 1, a double stranded DNA product comprising the DNA octahedron heavy chain sequence[3] (OHC) (with 5' Cy3 label (magenta star)) is obtained by PCR amplification of a synthetic gene, verified **b**) by 2% agarose gel electrophoresis (0.5X TBE). In step 2, the PCR product is captured on streptavidin magnetic beads through the (biotinylated) DNA template strand; (unbiotinylated) DNA OHC is denatured and eluted using sodium hydroxide. In step 3, the DNA template strand directs the synthesis of the 1,669 nt FANA OHC (with 5' Cy5 label (cyan star)) using an engineered XNA polymerase (PolD4K[4]), verified **c**) by Urea-PAGE (10% acrylamide, 1X TBE). NB: Like RNA, FANA oligonucleotides have a slower PAGE mobility than DNA, although mobility maybe be affected by incomplete denaturation of the extensive heavy chain secondary structure, which is also evident in samples of template DNA. Following step 3, a second streptavidin bead capture via the (biotinylated) DNA template enables the FANA OHC to be denatured, eluted and recovered by ethanol precipitation. **d**) Urea-PAGE (15% acrylamide, 1X TBE) analysis of purified DNA or FANA versions of the octahedron light chains.

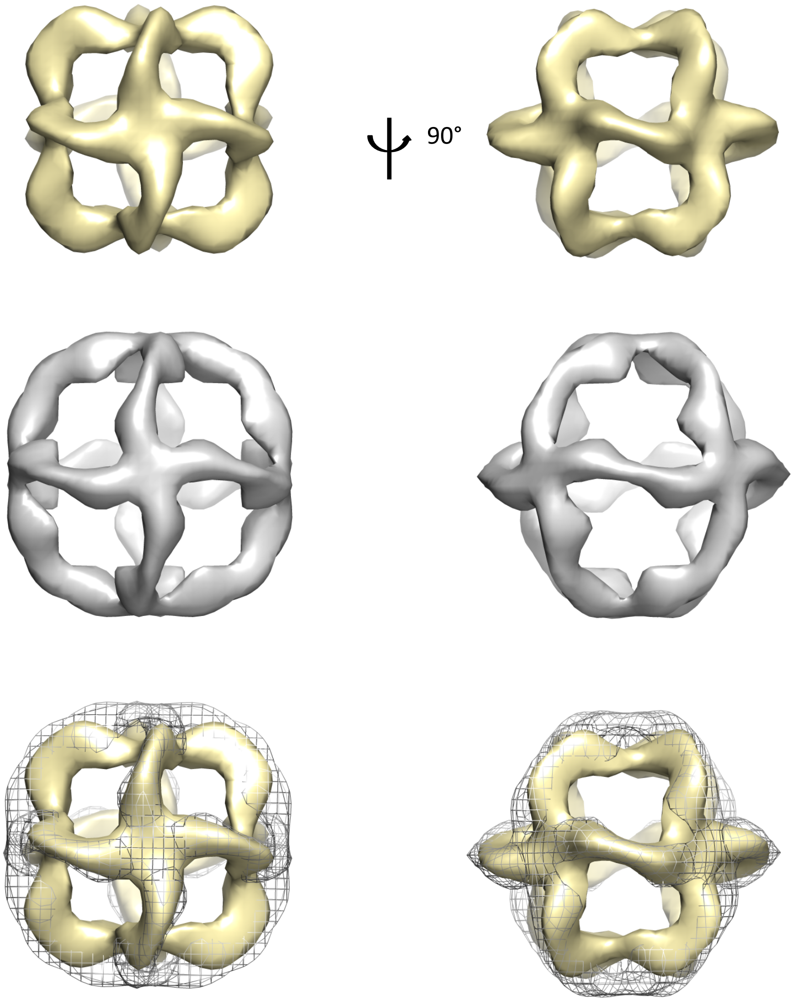

Figure S6: Alternative XNA (FANA) octahedron models.

Alternative models for the XNA (FANA) octahedron obtained form single-particle reconstruction using RELION 1.3[11] are shown in yellow (surface) and grey (surface and mesh). The volume enclosed in both cases corresponds to 450 Å^3. The two models are proposed to correspond to two slightly different conformations.

Table S1. Oligonucleotide sequences (5' -> 3') used for nanostructure synthesis.
FD sequence or its reverse complement is underlined. Red indicates RNA oligo.

Cy3_FD/ Cy5_FD / Thio_FD	5’ [Cy3] **or** [Cy5] **or** [5’ Thio Modifier C6 S-S] - CCCCTTATTAGCGTTTGCCA
RNA_FD	5’ [6FAM] – CCCCTTATTAGCGTTTGCCA
Tetra_Atemp<br>NB:<br>These templates contain the reverse complement of the sequences in Goodman *et al.*[1]	TACTATGGCGGCTCTTCTCGTGTAGCAAGCTGTAATGTTTCAGACTTAGGAATGTT<br>GGCAAACGCTAATAAGGG – 3’ [Biotin-TEG]
Tetra_Btemp	GTATTGGACCCTCGCATCTATTACAGCTTGCTACACTGTCAACTGCCTGGTGATAT<br>GGCAAACGCTAATAAGGG – 3’ [Biotin-TEG]
Tetra_Ctemp	GAAGAGCCGCCATAGTAGATTCCCACGTAGTGTCGTTTTATCACCAGGCAGTTGAT<br>GGCAAACGCTAATAAGGG – 3’ [Biotin-TEG]
Tetra_Dtemp	ATGCGAGGGTCCAATACAAACGACACTACGTGGGAAGCACATTCCTAAGTCTGAA<br>TGGCAAACGCTAATAAGGG – 3’ [Biotin-TEG]
Oct_lighttemp1	ATCATGCATTCGGCCACGGAGAATACGAGCAACGGTGCCGTGGCAAACGCTAATA<br>AGGG – 3’ [Biotin-TEG]
Oct_lighttemp2	CAGAGGCACCACTAGATGCGCTGATCTGGGTGGTTGCTATTGGCAAACGCTAATA<br>AGGG – 3’ [Biotin-TEG]
Oct_lighttemp3	TACCTATGCCACTTGCTACCAGTCGATTGGTGGCGTCTGGTGGCAAACGCTAATA<br>AGGG – 3’ [Biotin-TEG]
Oct_lighttemp4	TTCTCCGTCCACAGGGCCTGTTGTCGTAGGTGGAACCGACTGGCAAACGCTAATA<br>AGGG – 3’ [Biotin-TEG]
Oct_lighttemp5	ATTCGTAGCCACAACTGATTCTAGTCCCGGTGCACTGGGATGGCAAACGCTAATA<br>AGGG – 3’ [Biotin-TEG]
Oct_heavyRevP	5’ [Biotin-TEG] - CGATACATGGTGTGCTGC
Oct_heavytemp<br>NB:<br>The (-)strand (i.e. XNA synthesis template) is shown; this is the reverse complement of the sequence in Shi *et al.*[3], without restriction sites.	5’ [Biotin-TEG] -<br>CGATACATGGTGTGCTGCAAGGCAGAGTCCGGGACTAGAATCAGTTGTCCACTGG<br>CAGAAAACGCGCCGGCATGGTGCAGGTAGCGTCGGCATCCACGAAAACGTCCAT<br>TGCGACCATAGATGCAGGATCTCGGCGCGAAGCCGAGCTGGTGGCGCACCAAGT<br>AGCGACTAGTAGGAAAACCTCCAAGGCGCTCCGTGATGCGTCTCGCGCTCGGCA<br>AGCCTCGTCCCTGCGCGCTGGTCCGCCAGGTCCATGCAAAAGCAGCTAGATGGC<br>TCTGGCGCGCCGTCCCACGAGGCAATTCTGCCAGTGGTGTGGAAAACCACAGGC<br>TACGAATTCCCAGTGCAGGATAAAAATATCCACTCTGCCAATGTTCGGTCCTACGA<br>CAACAGGCCCTGTCCGATCCGGATGAACCCGGGCTGGACCGCCAGGACCATGCG<br>GCAGGGTGCAAAAGCAGGGACGCGCAGCCAGATGGCTCTAGCGCCCGGGAAGT<br>GGGGCTGTGGCGTTAGAGGTTGACAGCGTTGCGGAAAACCGGCATCATGTCTAG<br>GTTTAACCAGAGCGCCCCACAAGGCGGCGGCTGTACATCTACCTCCATAGAGTCT<br>CGCAAAAGCGGCCGTTTATGAACCTTGATGAACCCGCGCCGCCAACATCCGGATC

	GGTGTTAAAGCTTTAACAGGACGGAGAAGTCGGTTCCAGGCCTCTCGAGAGGCCA<br>CCGAACAAACGCCCCGTCGTGATGTAGATTGCGCCAGCTAGGCGGAAAACCGTC<br>AACATGGCTGACTTTACACCTTGGCGGGGCGAAGCAGCACAGGCCGAATGCATG<br>ATCGGCAGGAGCCATCAGGAAGGAGGGAAGTCCTCGTAGAGAGGCGTCTAGAGT<br>AGGAAAACCTCCGGTTGACGACAGTTTACGGCGAGCTCCCTCCAAGCACTTATCC<br>GGTGCTCCTGCAGGAGCAGGTGCCTCTGATAGCAACCAGGTGAGGATCCTCACC<br>ACGTCGATAACGTGAAGTCCAATCGACTGGTAGCAAGTCCTGATGCAAGAAACGG<br>CGCGACTCTACACTCCTCTCCAGAAGGACTCGCAAAAGCGGCTCGCTCTGAACTG<br>TAGTGAACCGGCGCGCCGAAGCGGGCACCTAGCGCCAGCAATCCACGTCACGAT<br>GCAAAAGCACCAAGGCGTGAAGTCATGGCTGTTGATGCCCGCAATCGCCGAAGA<br>CTCCATAGAGGTACATCTACAGCGCCAAAAGGCCGGGTTGATGAAGGTTTATGAA<br>CGGCTCGGCGAAATCTTGCATCAGGTGGCAAAAATGCCAGGCATAGGTACCAGAC<br>GCCAGGACAAAAATGTCCACTTCACGAAGGGGGACCAACGCGCTGAACCTCCAC<br>GGCCACATGGAAAACCAGCTCTGCGTGAACCTACAGCTGATGCGTCCCCCAAGCC<br>GACGACTAGTAGCGACTTGGCGCACCACCATGGAAAACCAGCGAGATGCGTCAC<br>GGCGCTCCTTGGCGTCGGCAAATCGACGTCCCAGATCAGCGCATCTAGTCCGGA<br>TAAGTGCAACGCGCCAGGATGCGTCGGCTACCGCGTCCATGCTCGAAAACGAAG<br>ATCCACGCTCTATGCGACCAATGGTGGCGCGAACCTGATGGCTCCACCACAAAAG<br>TGGTCCGTTGCTCGTATTCTCCGTCCATGTATCG<u>TGGCAAACGCTAATAAGGG</u>

Experimental Section

Materials

Oligonucleotides were obtained from IDT (USA); the 1.7 kb octahedron heavy chain sequence was obtained as a synthetic gene construct in pUCIDT (Amp). Triphosphates of HNA (hNTPs) and CeNA (ceNTPs) were synthesized and analyzed as described previously[4]. Triphosphates of 2'F-RNA (2'-F-dNTPs), FANA (2'-FANA-NTPs) and DNA (Illustra dNTPs) were obtained from Trilink BioTechnologies (USA), Metkinen Chemistry (Finland) and GE Life Sciences (USA), respectively.

Reagents were obtained from Sigma Aldrich (USA) unless specified otherwise.

XNA synthesis and purification

To prepare component strands[1] for XNA tetrahedra bearing DNA oligonucleotides, XNA syntheses were performed as described previously[4-6], using DNA oligonucleotides 'FD' (5' labeled with either Cy3 or, for preparation of GNP-linked strands, thio modifier) and 'Tetra_temp' A,B,C or D as primer and template, respectively. Engineered XNA polymerases were used as follows: PolD4K[4] for 2'fluoroarabino nucleic acids (FANA), Pol6G12[I521L][5] for hexitol nucleic acids (HNA), Pol6G12[4] for cyclohexene nucleic acids (CeNA) and PolTGK[7] for 2'fluorodeoxyribose nucleic acids (2'F-DNA). XNA strands (bound to biotinylated templates) were captured using Dynabeads MyOne Streptavidin C1 beads (Invitrogen/Life Technologies, USA), 10 µg beads / pmol XNA, in BWBS (10 mM Tris.HCl pH7.4, 1 M NaCl, 0.1 % v/v Tween20, 1 mM EDTA) for 1-2 hrs at room temperature or overnight at 4 ^{o}C. Beads were washed three times with excess BWBS and single-stranded XNA (ssXNA) eluted using 0.1 N NaOH at room temperature and neutralized with 1 M Tris.HCl pH 7.4. In order to prepare XNA-only tetrahedron components for the serum-stability assay, or to prepare octahedron light chains (using DNA oligonucleotides 'Oct_lighttemp' 1,2,3,4 or 5 as template), strands were synthesized using 'RNA_FD' primer, which was hydrolyzed after isolation of ssXNA by incubation in 0.7 N NaOH for 1 hr at 65 ^{o}C. XNA strands were purified by Urea-PAGE, as described previously[6].

To prepare the 1,669nt octahedron heavy chain, the synthesis scheme shown in Fig. S4 was used. PCR was performed using OneTaq Hot Start Master Mix (NEB, USA) using 1 µM primers ('Cy5_FD' and 'Oct_heavyRevP'), 5 ng/µl template (pUCIDT[Oct_heavytemp]) and cycling conditions 94 ^{o}C for 2 min, 35 X [94 ^{o}C for 1 min, 56 ^{o}C for 30 sec, 72 ^{o}C for 2 min], 72 ^{o}C for 5 min. PCR products were purified using Amicon 30,000 MW cut-off centrifugal filters (Merck Millipore, USA) and captured with streptavidin beads (1 µg beads / pmol PCR product) as described above. (+)strand (i.e. DNA octahedron heavy chain) was denatured and eluted using 0.1 N NaOH, then (biotinylated)(-)strand (i.e. octahedron heavy chain template) was recovered by three incubations in nuclease-free water (Qiagen GmbH, Germany) (beads at 10-20 mg/ml) at 80 ^{o}C for 2 mins. FANA synthesis was performed using the (-)strand (1.5 µM), primer 'Cy3_FD' (1 µM), 125 µM each fNTP and PolD4K (2 µM) in 1 X Thermopol buffer (NEB, USA) supplemented with 4 % ET-SSB (NEB, USA) and cycling conditions 50 ^{o}C for 30 min, 55 ^{o}C for 30 min, 65 ^{o}C for 2 hrs. Prior to addition of

polymerase and fNTPs, reactions were incubated at 95 °C for 5 min, then immediately transferred to ice for 2 min. After synthesis, reactions were incubated with streptavidin beads and FANA octahedron heavy chain denatured and eluted using NaOH, as described above.

To prepare XNA tetrahedra components labeled with gold nanoparticles (GNPs), Thio_FD-tagged XNA strands (1 µM) were incubated with 1 mM tris(2-carboxythyl)phosphine hydrochloride (TCEP) at 25 °C for 40 min, exchanged into 1X TBE (89 mM Tris-borate pH 8.3, 2mM EDTA) using 3,000 MW cut-off PES spin filters (Sartorius, Germany). Citrate-stabilized 10nm-diameter gold nanoparticles (GNPs)(Sigma Aldrich cat. no. 741957) were complexed with phosphine (BSSP) and conjugated to XNA strands according to the single-oligonucleotide functionalization method described by Taton[8]; 0.9 molar equivalent oligonucleotides to GNPs in 0.5X TBE, 25 °C for 16 hrs. GNPs coupled to single XNA strands were separated from poly- and unconjugated GNPs using agarose gel electrophoresis (at 4 °C), electro-eluted onto GF/B glass fiber (Whatman, UK) and recovered by filtration through a 0.45 µm Spin-X filter (Corning, USA).

MALDI-ToF mass spectrometry

Oligos were resuspended in water to 1 uM. 0.75 µl samples were spotted onto a maldi target followed by 0.75 µl 3-hydroxypicolinic acid. All mass spectrometric measurements were carried out in positive ion mode on an Ultraflex III TOF-TOF instrument (Bruker Daltonik, Bremen, Germany).

XNA nanostructure self-assembly

(Unconjugated) DNA and mixed XNA/DNA tetrahedra were prepared by mixing component strands A-D (1 µM each) in an equimolar ratio in 10 mM Tris.HCl pH 8.0, 1 mM EDTA, 10 mM $MgCl_2$ and annealed by incubation at 95 °C for 2 min then cooled at 0.1 °C/sec to 15 °C and incubated for 16 hrs, using a Tetrad thermocycler (BioRad, UK). XNA tetrahedra were prepared likewise, but used XTET buffer (10 mM Tris.HCl pH 8.0, 125 mM NaCl , 1 mM EDTA). GNP-conjugated tetrahedra were prepared using XTET buffer supplemented with 2mM $MgCl_2$. XNA tetrahedra were annealed as above, GNP-conjugates by incubation at 45 °C for 1 min then cooled at 0.1 °C/sec to 20 °C and incubated for 16 hrs.

DNA octahedra were prepared by mixing DNA versions of the heavy chain (100 nM) and light chains 1-5 (400 nM each) in OCT buffer (40mM EPPS pH 7.5, 50 mM NaCl, 10 mM $MgCl_2$), and annealed by incubation at 90 °C for 5 min, 65 °C for 20 min, 55 °C for 20 min, 45 °C for 20 min, 37 °C for 30 min, 15 °C for 1 hr then stored at 4 °C for at least 16 hrs. To prepare FANA octahedra, a two-step procedure was used, in which a partially-folded 'branched tree' structure[3] was formed first in the absence of magnesium, then folding completed by its addition. FANA heavy chain and FANA light chains 1-5 (concentrations as above) were mixed in 5 mM EPPS pH 7.5, 5 mM EDTA and incubated at 95 °C for 5 min, immediately followed by incubation on ice for 2 min then 25 °C for 10

min. OCT buffer was added to 1X final and mixtures incubated at 55 ^oC for 10 min then 25 ^oC for 10 min.

For EM samples, octahedra were purified using the method described by Bellot *et al.*[9]. Briefly, samples were separated on a dual-layer agarose gel (upper 2%, lower 4%) containing TBM buffer (45 mM Tris-borate, 2 mM $MgCl_2$) and GelStar stain (Lonza, Switzerland), run at 100 V at 4 ^oC. Bands corresponding to correctly-folded octahedra were electro-eluted into TBM supplemented with 30% sucrose in a well cut into the upper layer. Purified octahedra were exchanged into 1X OCT buffer using 3,000 MW cut-off PES spin filters (Sartorius, Germany).

Transmission electron microscopy of XNA structures

In order to image GNP-labeled tetrahedra, samples were deposited onto formvar-coated 400 mesh copper grids (Agar Scientific, UK). Grids were ionized by glow discharge, floated onto sample drops for 5 min, wicked with filter paper to remove excess sample. 'Quasi-3D'[2] images were collected at several holder α tilt angles using a Tecnai T12 electron microscope (FEI, USA) operating at 120 kV with an Orius detector (Gatan, USA) at a nominal magnification of x10,000.

For octahedra, samples were applied to glow-discharged carbon-coated Quantifoil grids (Quantifoil Micro Tools GmbH, Germany*)*, negatively stained with 2% (w/v) uranyl acetate and imaged on a Tecnai TF20 electron microscope (FEI, USA) operating at 200 kV with a F416 CMOS detector (TVIPS, Germany) at a nominal magnification of x50,000.

A set of 1,314 particles was assembled by manual picking using boxer, part of the EMAN2 package[10]. The particles were extracted into 200x200 boxes, coarsened, normalized and band-pass filtered between 150 and 25 Å. The 3D classification into 4 classes was carried out in RELION 1.3[11] using a smooth sphere as starting model and using octahedral symmetry during refinement. Indeed the analysis of the projection images indicates that views with 4-fold and other views with 2- and 3-fold rotational symmetry are present, consistent with the 432 octahedral point group symmetry. Two 3D maps are presented here which were selected based on connectivity between the density regions and overall shape.

Supplemental References

[1] R. P. Goodman, R. M. Berry, A. J. Turberfield, *Chem. Commun. (Camb.)* **2004**, 1372–1373.
[2] A. Stern, D. Rotem, I. Popov, D. Porath, *J. Phys.: Condens. Matter* **2012**, *24*, 164203.
[3] W. M. Shih, J. D. Quispe, G. F. Joyce, *Nature* **2004**, *427*, 618–621.
[4] V. B. Pinheiro, A. I. Taylor, C. Cozens, M. Abramov, M. Renders, S. Zhang, J. C. Chaput, J. Wengel, S. Y. Peak-Chew, S. H. McLaughlin, P. Herdewijn, P. Holliger, *Science* **2012**, *336*, 341–344.
[5] A. I. Taylor, V. B. Pinheiro, M. J. Smola, A. S. Morgunov, S. Peak-Chew, C. Cozens, K. M. Weeks, P. Herdewijn, P. Holliger, *Nature* **2015**, *518*, 427–430.
[6] A. I. Taylor, P. Holliger, *Nat. Protoc.* **2015**, *10*, 1625–1642.
[7] C. Cozens, V. B. Pinheiro, A. Vaisman, R. Woodgate, P. Holliger, *P. Natl. Acad. Sci. USA* **2012**, *109*, 8067–8072.
[8] T. A. Taton, *Curr. Proto.c Nucleic Acid Chem.* **2002**, *Chapter 12*, Unit 12.2.
[9] G. Bellot, M. A. McClintock, C. Lin, W. M. Shih, *Nat. Methods* **2011**, *8*, 192–194.
[10] G. Tang, L. Peng, P. R. Baldwin, D. S. Mann, W. Jiang, I. Rees, S. J. Ludtke, *J. Struct. Biol.* **2007**, *157*, 38–46.
[11] S. H. W. Scheres, *J. Mol. Biol.* **2012**, *415*, 406–418.
